# Progressive Freeze Concentration of Coconut Water and Use of Partial Ice Melting Method for Yield Improvement

**DOI:** 10.1155/2020/4292013

**Published:** 2020-02-24

**Authors:** J. A. E. C. Jayawardena, M. P. G. Vanniarachchy, M. A. J. Wansapala

**Affiliations:** Department of Food Science and Technology, Faculty of Applied Sciences, University of Sri Jayewardenepura, Gangodawila, Nugegoda, Colombo, Sri Lanka

## Abstract

Coconut water is a highly nutritious liquid food which is a by-product of the desiccated coconut industry. Freeze concentration is the most suitable concentration method for coconut water since the low-temperature operation for concentration does not deteriorate the original quality of coconut water. Suspension freeze concentration (SFC) and progressive freeze concentration (PFC) are the available FC methods, and SFC is a complex and expensive method compared with PFC. PFC is a novel freeze concentration technique to concentrate liquid food by using a simple system. The limitation of PFC is the lower product yield than SFC, and to overcome the problem, the partial ice-melting technique can be used. A simple cylindrical apparatus was used for PFC which consists of a sample vessel, agitator system, and a cooling bath (at −23°C ± 2°C temperature). The final concentration of the liquid product was directly affected by the apparatus agitator speed and sample vessel dipping speed. PFC agitator speed of 290 rpm and dipping speed of 1.3 cm h^−1^ were reported as the optimum operating conditions to achieve the highest concentration for the PFC apparatus used in this study. Using optimized agitation speed and dipping speed, coconut water was concentrated up to Brix 8.5° from the initial concentration of Brix 3.5°. PFC coconut water achieved 73.56% of total yield, 2.42 of concentration ratio, 0.7° of ice phase concentration, and 0.08 of effective partition coefficient. The partial melting technique was successfully explored by recovering initial ice fractions with high solute concentrations, and the total yield was improved up to 80%.

## 1. Introduction

Coconut (Cocos nucifera L.) water is an energizing, refreshing, and nourishing drink which is widely consumed in tropical countries [1]. According to Prades et al. [2], coconut water signifies between 15% and 30% of the weight of the nut. The amount of coconut water that can be harvested from each nut is about 300 mL. The composition of coconut water accounts for 95% of water and 4% carbohydrates and 0.1% fat; in addition to that, coconut water is enriched with amino acids, vitamin C and B complex vitamins, and minerals.

Coconut water is a by-product of desiccated coconut (DC) industry. About 10 years ago, coconut water from DC was a waste. The growing demand for coconut water is high as people are concerning the health benefits it offers [3, 4].

In Sri Lanka, the DC industry exports coconut water as a beverage just after going through a pasteurization/sterilization process without concentrating. Therefore, large volumes of coconut water have to be handled during exporting which is a waste of money on transportation and storage. The possible solution to this problem is to reduce the volume of the coconut water by concentration.

The main objective of concentration is to reduce packaging, transportation, and storage cost and to increase the stability of the products [5]. There are three methods of concentration that can be used in liquid food industry. Those are evaporative concentration, membrane concentration, and freeze concentration (FC) [6].

Among these, the evaporative concentration is the oldest, best developed, and economically feasible liquid concentration method, widely used up to date. But the quality of the concentrated liquid food is poor with loss of flavors, color, and nutrients.

Membrane concentration is an alternative method to replace evaporative concentration. The generally used membrane method for removing water from liquid food is reverse osmosis (RO). Due to the possibility of operation at room temperature, it causes less thermal damages with increased aroma retention than evaporative concentration. The limitations of this method are membrane fouling, difficulties in cleaning, requirement of regular changing of the expensive membrane, and incapability to achieve high concentration levels like in evaporation [7].

According to many published research studies, freeze concentration (FC) can be selected as the best concentration method in terms of maintaining original quality in the concentrated product [5, 7–9].

There are different types of freeze concentration methods available, suspension freeze concentration (SFC), progressive freeze concentration (PFC), and block cryoconcentration [6, 10]. The yield of concentration in SFC is high with high purity of ice crystals. It requires a special equipment to separate ice from the mother solution because of the large surface area of large number of small ice crystals. Therefore, this method requires a complicated system which makes SFC the most expensive method of the other concentration methods [11].

BFC is a simple FC method which follows freezing-thawing method and requires improvements for the future developments. In BFC, the whole liquid food is frozen into a block and then partially thawed to get the concentration fraction. This method is applied in PFC to increase the total yield of the concentrated product [10].

PFC is a type of FC that progressively produces a single ice crystal layer by layer on a cold surface until it forms a large single ice block. Concentration happens by removing water molecules from the liquid food by attaching to the progressively growing single ice front. Using proper operating conditions with a low crystal growth rate, relatively high concentration efficiency can be achieved by PFC [12]. Due to easy separation of concentrated solution from a single ice block, PFC requires a simple system, and it is a low-cost technology than conventional FC methods [11].

Therefore, this novel process requires a simple operating system with a lower cost that can be applied to many liquid foods [13].

The PFC method is still at a developing stage, and many studies have been conducted to develop a new design of PFC systems with a higher productivity and efficiency [11]. Conferring to Miyawaki et al. [14] and Shirai et al. [15], the tubular ice system is an effective scale-up system for PFC that increases the efficiency at a slow ice growth rate and at a high mixing speed.

The major problem in PFC system is the decrease in yield with the increase in the concentration. To overcome this problem, partial ice-melting technique can be used, where the principle of the freezing-thawing technique was successfully applied. The purity of ice crystals produced from the PFC is low compared with that from the SFC. But solutes can be recovered by partially melting the formed ice crystal which helps to improve the total yield [12]. The first melted ice fractions have higher concentrations than the later fractions. By collecting higher concentration fractions, the total yield of the PFC can be increased [16, 17].

In this research, a small cylindrical apparatus was used for progressive freeze concentration of coconut water, and partial ice melting method was used to improve the total yield of the final product. Optimum operating conditions of the apparatus were found using standard sucrose solutions. The key objective of this study is to concentrate coconut water while preserving the original quality by low-temperature operation using a simple PFC system.

## 2. Materials and Methods

### 2.1. Progressive Freeze Concentration Setup

**Figure 1:**
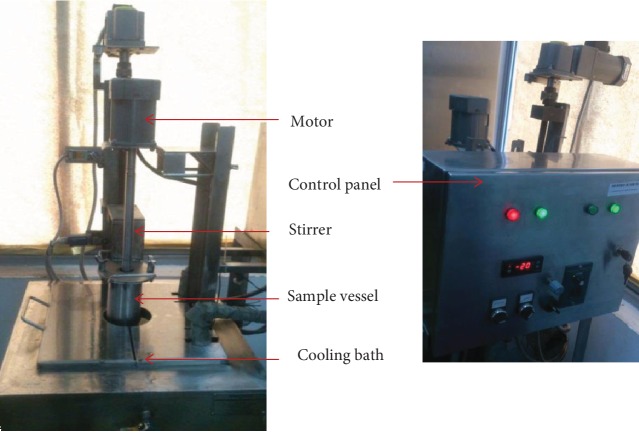
Constructed laboratory scale PFC setup.

**Figure 2:**
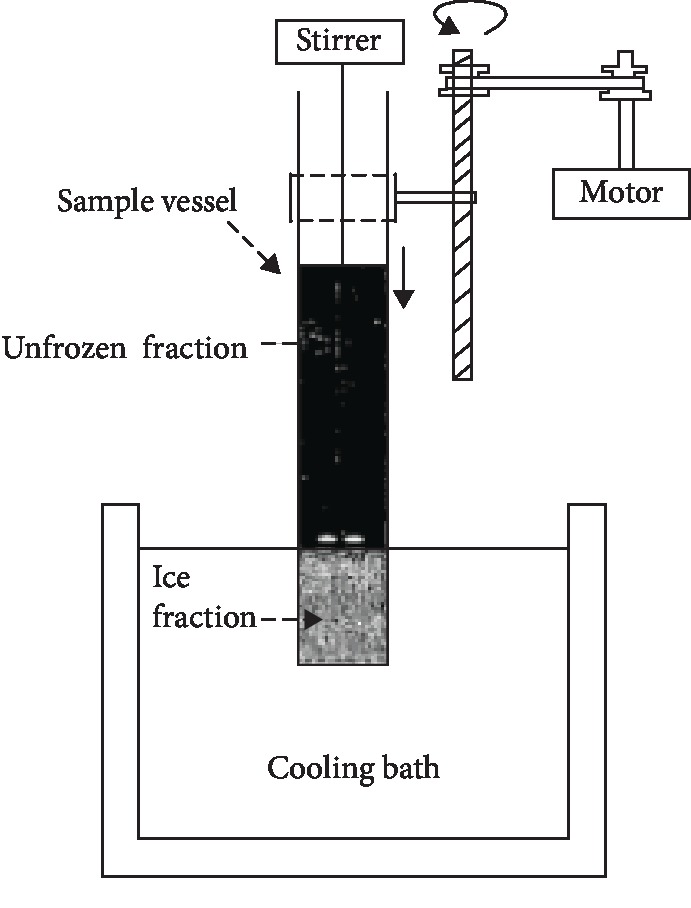
The sketch diagram for the PFC setup; source: [6, 18].

The simple cylindrical apparatus as shown in Figure 1 was constructed at Advanced Engineers (Pvt) Ltd., Delkanda, Nugegoda, according to the schematic design (Figure 2) from literature [6, 18]. The setup mainly consists of cylindrical stainless steel (AISI 304) sample vessel equipped with a stirrer and stainless steel ethylene glycol/water cooling bath. The sample vessel (100 mm diameter, 300 mm height) was plunged into the cooling bath with a constant slow speed. The stirrer consists of a shaft and a two-blade propeller (45 cm diameter). The coolant used was ethylene glycol (Daejung Chemicals, South Korea). About 83.33% *V*/*V* ethylene glycol was mixed with distilled water 16.67% *V*/*V* to achieve the minimum temperature of the cooling bath at −23°C ± 2°C, and the temperature of the coolant was maintained by the control panel and the compressor unit (NTZ 048; Danfoss Maneurop, China).

### 2.2. The Operational Procedure of PFC Setup

The PFC setup was connected to the power and kept overnight (24 h) to cool down the ethylene glycol until the ethylene glycol bath temperature reaches its minimum temperature (−23°C ± 2°C). About 2 mL of distilled water was added to the sample vessel, and the bottom of the vessel was allowed to be cooled by glycol bath to produce initial seed ice layer in order to overcome the supercooling effect. Supercooling disrupts the concentration process and increases the solute contamination in the ice phase [6, 18]. The precooled sample was added to the sample vessel closely reaching to its freezing point to avoid the melting of the formed seed ice layer. The sample vessel was slowly dipped into the cooling bath using an inverter system with continuous agitation at the liquid-ice interphase. Vessel dipping speed and agitator speed were controlled by the control panel.

### 2.3. Data Analysis

The solute separation efficiency at ice-liquid phase is defined by the apparent partition coefficient value. It is a factor to describe the efficiency in a progressive freeze concentration process.

Equation (1) shows the calculation of apparent partition coefficient.

(1) $$\begin{array}{c} K_{\text{app}}=C_{\text{s}}/C_{\text{L}}, \end{array}$$

where *K*_app_ is apparent partition coefficient, *C*_s_ is concentration of the ice phase, and *C*_L_ is solute concentration of the liquid phase.

The total yield of the concentrated product based on Brix was calculated by Equation (2).

Equation (2) shows the calculation of yield in PFC liquid food.

(2) $$\begin{array}{c} \text{Yield}=\frac{C_{\text{conc}}\times V_{\text{conc}}}{\left(C_{\text{conc}}\times V_{\text{conc}}\right)+\left(C_{\text{ice}}\times V_{\text{ice}}\right)}, \end{array}$$

where *C*_conc_ and *C*_ice_ are a concentration of concentrated liquid phase and ice phase and *V*_conc_ and *V*_ice_ are volumes of those phases. *V*_ice_ was measured after melting the whole ice phase.

### 2.4. Optimization of the PFC Setup

Standard sucrose solutions of 1° Brix and 2° Brix were prepared using sucrose standard (Sigma-Aldrich, USA) and used as the samples for optimization of the progressive freeze concentration process. The concentration of the samples was interpreted by Brix value and measured using a digital refractometer (ATAGO PAL-1, Japan; 0-53% Brix) at room temperature (29°C ± 0.5°C). Sample volume was 500 mL for each experiment.

#### 2.4.1. Optimization of Agitation Speed

The prepared samples were precooled in order to cool down the sample up to its freezing point. Then, the PFC setup was operated according to the operational procedure. The experiment was repeated for 7 different agitator stirring speeds 72.5 rpm, 145 rpm, 217.5 rpm, 290 rpm, 362.5 rpm, 435 rpm, and 507.5 rpm, respectively. After 5-hour operation, the liquid phase and ice phase were collected separately. Agitator speeds vs. concentration of the liquid phase (*C*_L_) and concentration of the ice phase (*C*_s_) graphs were plotted according to results obtained in the experiments.

Apparent partition coefficient (*K*_app_) of solute between the ice phase and liquid phase in progressive freeze concentration was calculated applying the obtained results to Equation (1) [18].

#### 2.4.2. Optimization of Sample Vessel Dipping Speed

The experiment was repeated using 8 different dipping speeds 1.3 cm h^−1^, 2.3 cm h^−1^, 2.7 cm h^−1^, 3 cm h^−1^, 3.4 cm h^−1^, 3.7 cm h^−1^, 4.1 cm h^−1^, and 4.7 cm h^−1^, respectively. The sample was agitated using optimized agitator speed. The sample vessel dipping speed vs. concentration of liquid phase (*C*_L_) and concentration of ice phase (*C*_s_) graphs was plotted, and the apparent partition coefficient (*K*_app_) was calculated using the obtained results in the experiments.

### 2.5. Progressive Freeze Concentration of Coconut Water

The fresh matured coconut water was taken from the coconut water chilling center, St. Joseph DC Mills, Watinapaha, Sri Lanka. To obtain the liquid endosperm (coconut water) of mature coconut, mesocarp (fibrous husk) and endocarp (hard nutshell) were removed hygienically. Collected coconut water was immediately chilled to 2°C-4°C temperature to avoid probable development of any contaminants. Chilled mature coconut water was filtered using a muslin cloth and taken to the experiment.

Coconut water was concentrated by the PFC setup, and the best concentration was achieved using optimized agitator stirring speed and optimized vessel dipping speed. According to Miyawaki et al. [13], the yield of concentrate was calculated by Equation (2).

### 2.6. Partial Ice Melting for Yield Improvement

**Figure 3:**
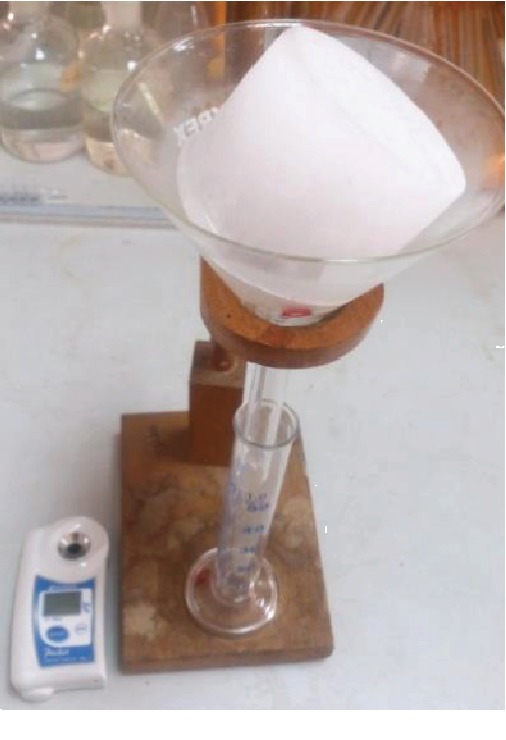
Partial melting of formed ice fraction of PFC; melted volume collected in 10 mL fractions.

The Partial ice melting method was conducted to improve the yield of concentrate according to the procedure of Miyawaki et al. [12]. The formed ice fraction of the progressive freeze concentration was separated from the concentrate and placed on a funnel and kept for melting at room temperature (29°C ± 0.5°C) as shown in Figure 3 Melted ice volume was collected as 10 mL fractions. Brix values of each fraction were recorded using a digital refractometer, and the maximum achievable concentration of the initial fractions was evaluated.

### 2.7. Statistical Analysis

All the analysis were carried out in five replicates and was reported as mean ± standard deviation at *p* > 0.05 level of significance.

The collected data was analyzed by using Minitab 17 statistical package. For the graphical representation of the data, Minitab 17 package was used.

## 3. Results and Discussion

### 3.1. Progressive Freeze Concentration

**Figure 4:**
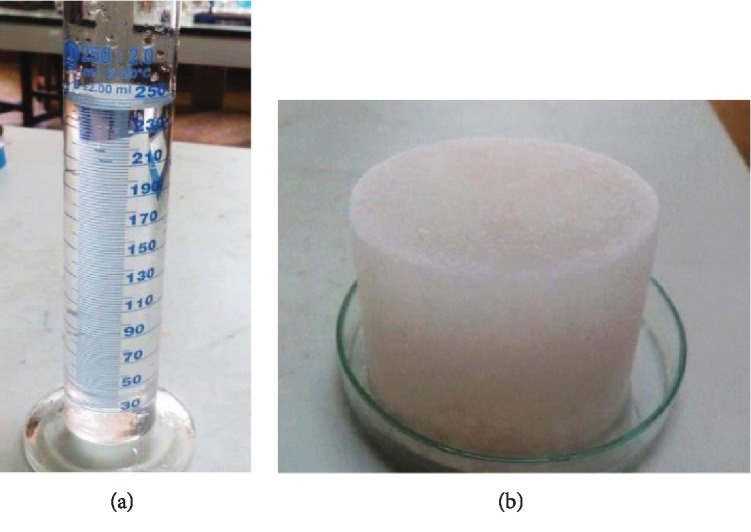
(a) Unfrozen fraction (liquid phase) and (b) frozen fraction (ice phase).

In PFC, ice is forming layer by layer from the bottom of the sample vessel until a single ice block is formed. After 5-hour operation, the sample vessel was removed from the system, and unfrozen fraction (Figure 4(a); liquid phase) and the frozen fraction (Figure 4(b); ice phase) were collected separately.

### 3.2. Optimization of the PFC Setup

#### 3.2.1. Optimization of Agitation Speed

**Figure 5:**
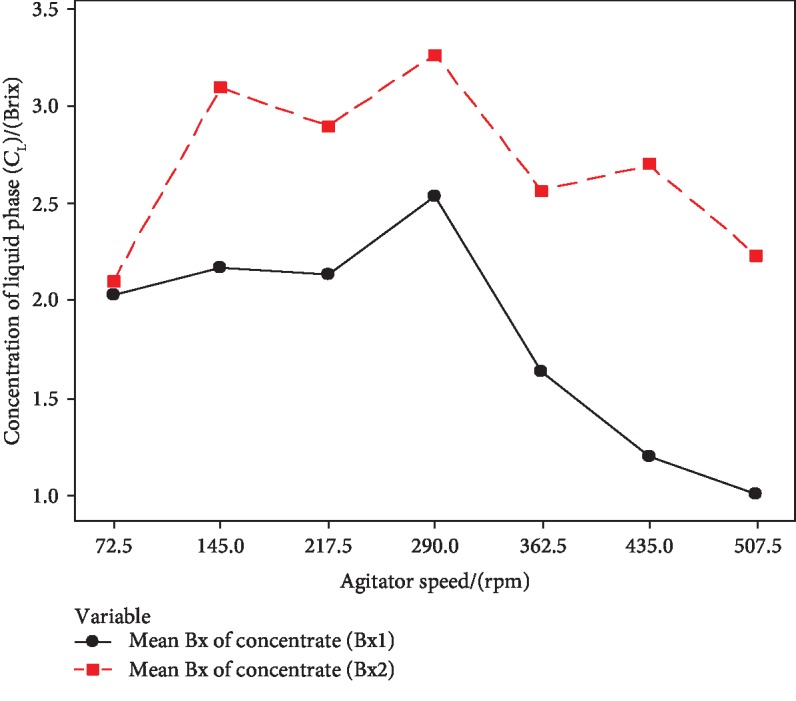
Effect of agitator speed on the concentration of the liquid phase (*C*_L_).

**Figure 6:**
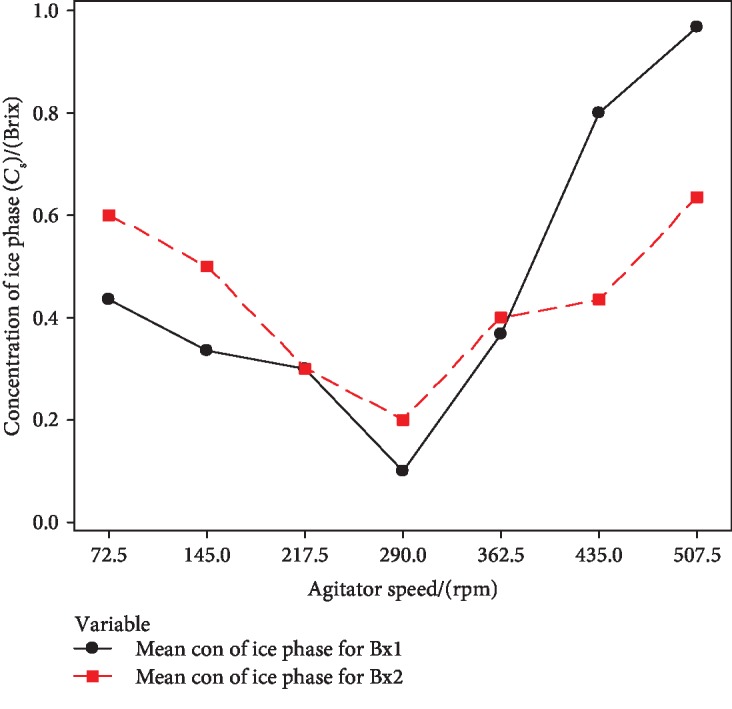
Effect of agitator speed on the concentration of ice phase (*C*_S_).

The standard Brix solutions (1° and 2° Brix) were progressively freeze concentrated using the PFC setup. The experiment was repeated for 7 different agitator speeds, and these agitator values were taken based on the capacity of the offered gear motor. The concentration of the liquid phase (*C*_L_) and concentration of the ice phase (*C*_S_) were interpreted by the Brix value and were measured using a digital refractometer. Figures 5 and 6 interpreted the effect of agitator speed on *C*_L_ and *C*_S_, respectively.

According to Figure 5, *C*_L_ was increased with the agitator speed up to 290 rpm and there was an abrupt decrease at 217.5 rpm for both 1° Brix and 2° Brix solutions. The highest concentration was achieved at 290 rpm, and thereafter, *C*_L_ was gradually decreased with mixing speed.

Figure 6 shows a gradual decrease of *C*_s_ with the agitator speed up to 290 rpm, and afterward, *C*_s_ was increased for both 1° Brix and 2° Brix solutions. The lowest ice phase concentration was achieved by 290 rpm mixing speed. In PFC, the mixing speed at the ice-liquid interface is directly affected by the concentration of the PFC product and purity of the ice phase [13, 16, 18]. Theoretically, the concentration efficiency of the liquid phase should be increased with mixing speed and the purity of ice also increased. Shirai et al., Miyawaki et al., Ojeda et al., and Muñoz et al. stated that stirring speed positively affected to the concentration of the unfrozen fraction [14, 15, 19, 20]. According to the obtained results, increasing agitator speed adversely affected the concentration process after 290 rpm. One possible reason could be uneven temperature distribution in the ethylene glycol cooling bath of constructed PFC setup. Another reason could be high mixing speed which is resulting in the generation of heat above the ice layer and increases the temperature of the liquid phase, and this may adversely affect to the pure ice crystal formation in PFC. The higher velocity of agitation of the stirrer (45 mm of propeller blade agitator) could be affected to generate some heat due to the limited space of the vessel volume (100 mm diameter, 300 mm height) of the constructed PFC setup. Further analysis is needed for the increment of sample volume to overcome the adverse effect by higher agitation.

**Figure 7:**
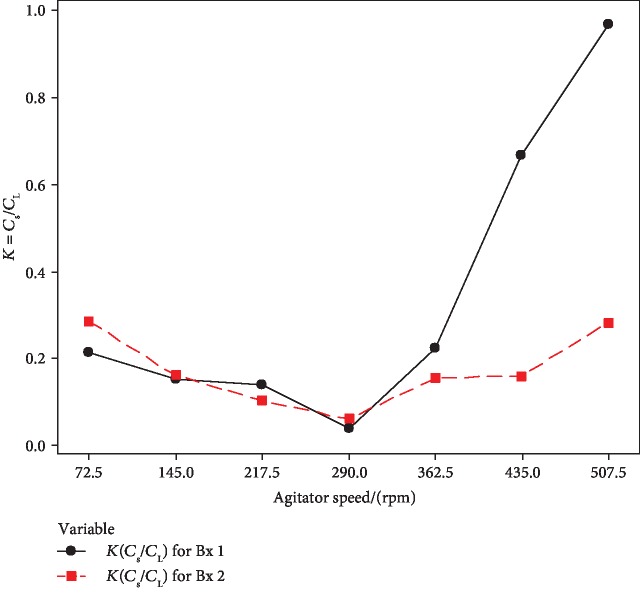
Change of apparent partition coefficient (*K*_app_) with PFC agitator speed (rpm).

Using Equation (1), the apparent partition coefficient was calculated.  Figure 7 shows the change of the apparent partition coefficient against the PFC agitator speed.

The apparent partition coefficient is a factor to describe the solute separation efficiency at ice-liquid interphase in PFC [21]. Using proper operating conditions, the concentration efficiency can be increased [16]. Analyzing the apparent partition coefficient is important, in order to optimize the mixing (agitator) speed. The experimentally apparent partition coefficient (*K*_app_) should be decreased with agitator stirring speed. But here, *K*_app_ value is gradually decreased up to 290 rpm and then increased with the agitator speed. The solute incorporation into the ice phase is gradually increased after 290 rpm due to the increase of heat generation above the ice layer happening after 290 rpm. Analyzing all the results, 290 rpm was selected as the optimum agitator speed for constructed lab scale PFC setup.

#### 3.2.2. Optimization of Sample Vessel Dipping Speed

**Figure 8:**
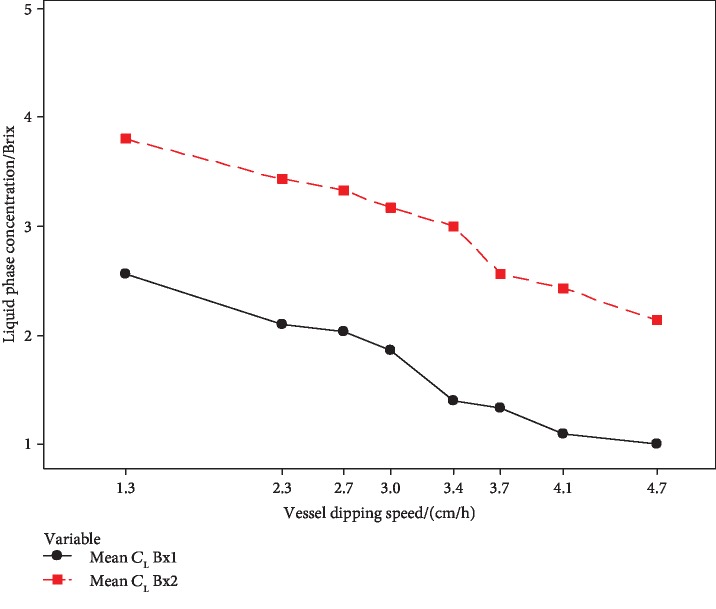
Effect of sample vessel dipping speed on the concentration of liquid phase (*C*_L_).

**Figure 9:**
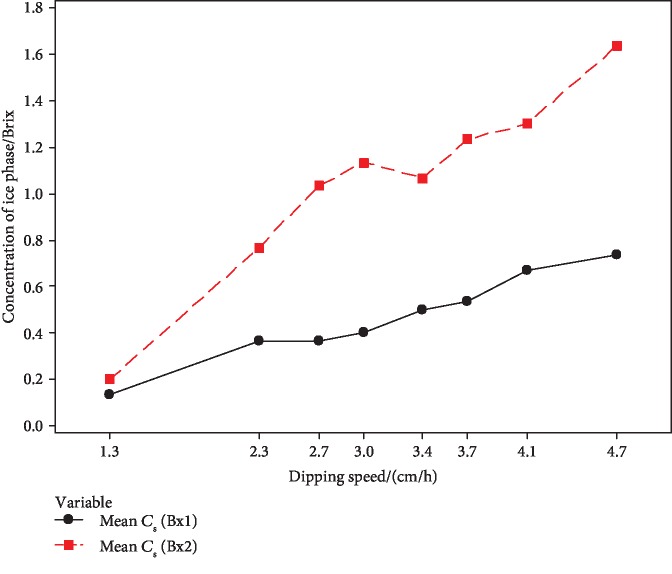
Effect of sample vessel dipping speed on the concentration of ice phase (*C*_s_).

For the optimization of sample vessel dipping speed, 8 different vessel dipping speeds were taken. Figures 8 and 9 represent the effect of the vessel dipping speed in a change of concentration of the liquid phase (*C*_L_) and concentration of the ice phase (*C*_S_).

The concentration of the unfrozen fraction was gradually decreased with the vessel dipping speed (Figure 8). The highest concentration was achieved by the lowest vessel dipping speed (1.3 cm h^−1^) of the constructed PFC setup.

According to Figure 9, concentration of the frozen fraction gradually increased with the vessel dipping speed. The increasing sample vessel dipping speed was adversely affected by the concentration of the liquid phase (*C*_L_), and it positively affected the ice phase concentration (*C*_S_). The slow-moving speed of ice front into the cooling bath influenced the formation of pure ice.

**Figure 10:**
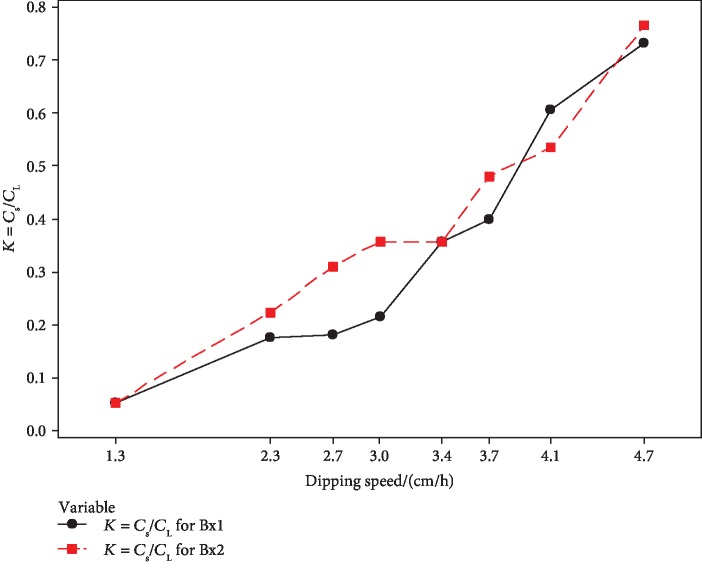
Effect of the dipping speed with the apparent partition coefficient (*K*_app_).

The apparent partition coefficient (*K*_app_) was calculated to analyze the relationship between *C*_s_ and *C*_L_ with the increasing dipping speed.  Figure 10 shows the effect of dipping speed with the apparent partition coefficient value (*K*_app_).

The mean *K*_app_ value is increased with the effect of dipping speed. The lowest *K*_app_ value represents the highest concentration of the unfrozen fraction and the highest purity level of the ice fraction. According to the results, the lowest speed, 1.3 cm h^−1^, was selected as the optimum dipping speed for the constructed PFC setup. Slow dipping speed influences the growth of pure ice in the progressive freeze concentration.

The constructed PFC setup was optimized to collect the maximum achievable concentration of liquid foods using standard sucrose solution.

Using an apparent partition coefficient, the optimized conditions were evaluated. The optimized agitator speed was 290 rpm and optimized dipping speed was 1.3 cm h^−1^.

### 3.3. Progressive Freeze Concentration of Coconut Water

Coconut water was progressively freeze concentrated using the PFC setup and concentrated liquid fraction and ice fraction were collected separately.  Table 1 shows the results of concentrated coconut water products. Initial volume (*V*_0_) for each sample was 300 mL.

Using optimum operating conditions, coconut water was concentrated up to 8.5° from the initial concentration of 3.5°. The total yield, concentration ratio, and apparent partition coefficient for PFC coconut water were 73.56 ± 0.10, 2.42, and 0.08, respectively. Apparent partition coefficient (*K*_app_ = *C*_s_/*C*_L_) is a parameter to describe the solute separation efficiency at the ice-liquid interphase in PFC [16]. Since coconut water has a lower effective partition coefficient value, the efficiency to PFC is high.

Liu et al. [18] and Miyawaki et al. [6] state that PFC was effective for low initial concentration liquid food with the lowest osmotic pressures. Freezing is affected with solute concentration, osmotic pressure, presence of electrolytes, and the viscosity of the liquid. Therefore, in PFC, those factors are affecting the final concentration and yield of the PFC product. However, the yield of the PFC product will be decreased with the high solute concentration of the liquid. Therefore, the liquids with low concentrations and low osmotic pressure are the most suitable for PFC.

### 3.4. Partial Ice Melting for Yield Improvement

The major drawback in progressive freeze concentration is the decrease in yield with an increase in the concentration. To overcome the problem, a partial ice-melting technique can be used, where the principle of the freezing-thawing technique was successfully applied. The first melted ice fractions have higher concentrations than the later fractions. By collecting higher concentration fractions, the total yield of the PFC can be increased [13, 16].

The partial melting method was applied to all the ice fractions collected from PFC coconut water, and the yield was calculated according to Equation (2).

**Figure 11:**
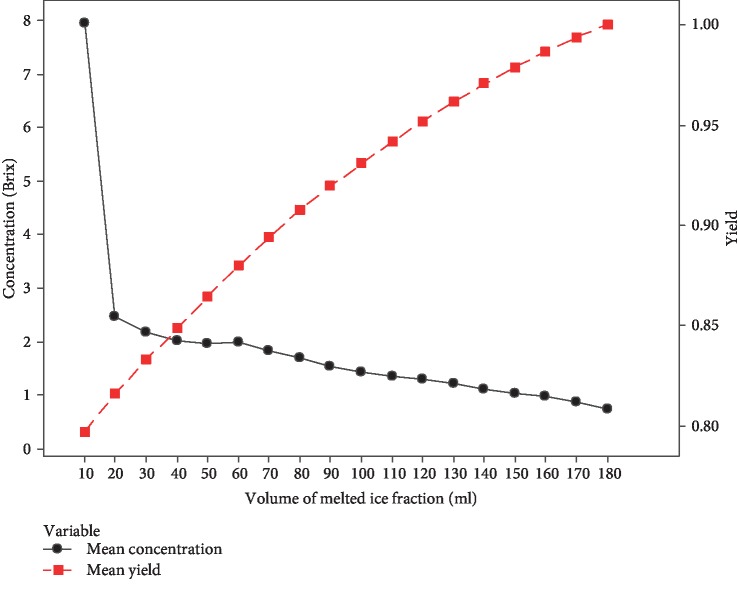
Yield improvement of PFC coconut water by partial melting of ice fraction (^∗^mean concentration and mean yield of replicate changes with the melted ice volume).

Figure 11 shows the change in concentration and yield (based on Brix) with the melted ice volume of PFC coconut water (mean concentration and mean yield of replicate changes with the melted ice volume were illustrated). The concentration was decreased gradually, and the yield was increased with the melted ice fractions. From Figure 11, it was identified that the first 10 mL melted ice fraction has a higher Brix value than the concentrated coconut water, thereby recovering the first 10 mL fraction; the yield can be improved up to 80 ± 0.10% from the initial yield of 73.56 ± 0.10%. The melted fractions can be added to the next batch to improve the total yield of the PFC product. The partial melting technique was important in progressive freeze concentration of liquids with higher solute concentration. Thus, the higher concentration increases the amount of solute incorporation to the ice phase. Recovering the melted ice fractions was effective to improve the total yield of the final product. Also, the recovering fractions could be used for the next batch of PFC process. In the study, exploring a partial melting technique to PFC proved that the total yield of the PFC product can be improved to a positive level.

## 4. Conclusion

The progressive freeze concentration setup consists of a simple system which can be used for high-quality liquid food concentration. The constructed PFC setup was optimized to collect the maximum achievable concentration of liquid foods using standard sucrose solution. Using an apparent partition coefficient, the optimized conditions were evaluated. The optimized agitator speed was 290 rpm and optimized dipping speed was 1.3 cm h^−1^.

Using optimized operating conditions, coconut water was concentrated from Brix 3.5° to Brix 8.5° by progressive freeze concentration method. PFC coconut water achieved 73.56% of yield, 2.42 of concentration ratio, 0.7° of ice phase concentration, and 0.08 of apparent partition coefficient.

The decrease in yield with an increase in the concentration was the major problem in progressive freeze concentration. Partial ice melting is a technique, where the principle of the freezing-thawing technique was successfully applied. By recovering initial (10 mL) ice fractions with high solute concentrations, the total yield can be improved up to 80%. Therefore, in this study, partial ice melting technique was successfully explored to improve the effectiveness of the PFC process.

PFC is the most suitable method to concentrate coconut water without deteriorating its original quality using a simple system which requires low cost than SFC. Therefore, PFC is a promising method for concentrating coconut water.

## Figures and Tables

**Table 1:** Results of PFC coconut water. *C*_conc_ and *C*_ice_ are the concentrations of the concentrated liquid phase and ice phase, and *V*_conc_ and *V*_ice_ are volumes of those phases.

Liquid food	*C*_initial_ (° Brix)	*V*_conc_ (mL)	*C*_conc_ (° Brix)	*V*_ice_ (mL)	*C*_ice_ (° Brix)
Coconut water	3.5 ± 0.08	120.4 ± 3.29	8.5 ± 0.53	179.6 ± 3.29	0.7 ± 0.24

^∗^ *Mean values of replicates were interpreted in results and SD (Standard deviation) is shown as ± value.*

## Data Availability

The numerical data used to support the findings of this study are included within the article.
